# *Neodendryphiella*, a novel genus of the Dictyosporiaceae (Pleosporales)

**DOI:** 10.3897/mycokeys.37.27275

**Published:** 2018-07-26

**Authors:** Isabel Iturrieta-González, Josepa Gené, Josep Guarro, Rafael F. Castañeda-Ruiz, Dania García

**Affiliations:** 1 Unitat de Micologia, Facultat de Medicina i Ciències de la Salut and IISPV, Universitat Rovira i Virgili, Reus, Spain Universitat Rovira i Virgili Reus Spain; 2 Instituto de Investigaciones Fundamentales en Agricultura Tropical “Alejandro de Humboldt” (INIFAT), 17200, La Habana, Cuba Instituto de Investigaciones Fundamentales en Agricultura Tropical “Alejandro de Humboldt” Habana Cuba

**Keywords:** *
Dendryphiella
*, Ascomycota, Phylogeny, Taxonomy

## Abstract

In a survey of soil and herbivore dung microfungi in Mexico and Spain, several dendryphiella-like species were found. Phylogenetic analyses based on ITS and LSU sequences showed that these fungi belonged to the family Dictyosporiaceae (Pleosporales) and represent an undescribed monophyletic lineage distant from *Dendryphiella*. Therefore, the genus *Neodendryphiella* is proposed to accommodate three new species, *N.mali*, *N.michoacanensis* and *N.tarraconensis*. The novel genus shares morphological features with *Dendryphiella* such as differentiated conidiophores and polytretic integrated conidiogenous cells, that produce acropetal branched chains of conidia. *Neodendryphiella* differs in the absence of nodulose conidiophores bearing conidiogenous cells with pores surrounded by a thickened and darkened wall, typical features in the conidiogenous apparatus of *Dendryphiella*. In addition, the phylogenetic and morphological analysis of several reference strains of different *Dendryphiella* species, available for comparison, support the proposal of *D.variabilis***sp. nov.**, which mainly differs from the other species of the genus by having conidia up to 7 septa and highlight that *D.vinosa* and *D.infuscans* are obscure species that require further taxonomic review.

## Introduction

In an ongoing survey of asexual microfungi from soil and herbivore dung, several interesting specimens morphologically consistent with *Dendryphiella* were found from samples collected in Mexico and Spain. *Dendryphiella* is a dematiaceous hyphomycete proposed by Bubák and Ranojevič (Ranojevič 1914) and typified with *D.interseminata*, which is currently considered a synonym of *D.vinosa* (Reisinger 1968). *Dendryphiellavinosa* is a saprobic fungus commonly found on plant debris, especially on the decaying herbaceous stems of several plants (Ellis 1971, Mercado Sierra et al. 1997). The genus is characterised by pigmented conidiophores, with terminal or intercalary polytretic conidiogenous cells, with dark scarring on the nodose swellings, producing acropleurogenous, solitary or catenate conidia, which are commonly multi-septate and cylindrical with rounded ends (Ellis 1971). Although Index Fungorum and MycoBank list 17 taxa in *Dendryphiella*, a recent review of literature reported only 12 species are accepted, including the newly proposed *D.fasciculata* (Liu et al. 2017). *Dendryphiellapitsanulokensis* is the latter species added to the genus (Hyde et al. 2018). Previous phylogenetic studies, conducted mainly from sequence data of the 18S nrDNA (SSU), 28S nrDNA (LSU) and the internal transcribed spacer (ITS) nrDNA regions, showed that the marine species *D.arenariae* and *D.salina* were phylogenetically distant from the type *D.vinosa* and related to the Pleosporaceae (Gareth Jones et al. 2008, Suetrong et al. 2009). Both species were therefore moved to the genus *Paradendryphiella* (Woudenberg et al. 2013) and, more recently, *D.vinosa* was included in the family Dictyosporiaceae (Tanaka et al. 2015, Boonmee et al. 2016). However, DNA sequence data for *Dendryphiella* species is very limited to create a robust taxonomy for the genus. Only LSU and/or ITS sequences of *D.eucalyptorum*, *D.fasciculata*, *D.paravinosa*, *D.pitsanulokensis* and *D.vinosa* are available (Crous et al. 2014, 2016, Liu et al. 2017, Hyde et al. 2018). In addition, with the exception of the first four mentioned, there is no ex-type culture of other species of this genus and only reference strains of *D.vinosa* and *D.infuscans* are available in public collections for comparison.

Despite the similarity of our soil isolates to *Dendryphiella*, a preliminary study revealed that they showed a low sequence relationship with members of this genus. On the other hand, they were closely related to the strain CBS 139.95 of Diplococcium (Di.) asperum, which was proven to be related to the Dictyosporiaceae (Shenoy et al. 2010, Boonmee et al. 2016). It is well known that the genus *Diplococcium* is highly polyphyletic, with species distributed across different classes of the Ascomycota, with its type species, *Di.spicatum*, being related to the Helotiales in Leotiomycetes (Shenoy et al. 2010, Hernández-Restrepo et al. 2017).

The aim of the present study was to resolve the taxonomy of these dendryphiella-like fungi which, based on analysis of the ITS and LSU loci, might represent a new genus in Dictyosporiaceae.

## Material and methods

### Sampling and fungal strains studied

Soil and dung samples collected in different geographical regions (Mexico and Spain) were studied using the wood baiting technique, moist chambers and dilution plating method according to Calduch et al. (2004). Using the first two techniques, we found three interesting dendryphiella-like fungi, which were isolated on Potato Dextrose Agar (PDA; Pronadisa, Madrid Spain) and incubated at room temperature in the dark. Additionally, six strains from the Westerdijk Fungal Biodiversity Institute, Utrecht, The Netherlands (CBS), which corresponded to *D.vinosa* (CBS 117.14, CBS 118716, CBS 121797 and CBS 584.96), *D.infuscans* (CBS 381.81) and *Di.asperum* (CBS 139.95) were included in the study for morphological and sequence comparison (Table 1).

**Table 1.** Species included in this study, their origin and GenBank accession numbers.

**Species**	**Original identification**	**Strain number^1^**	**Country**	**Genbank accession no.^2^**	
				**ITS**	**LSU**
* Aquaticheirospora lignicola *		RK-2006a (T)	Thailand	AY864770	AY736378
* Cheirosporium triseriale *		HMAS 180703 (T)	China	EU413953	EU413954
*** Drechslera biseptata ***	*** Dendryphiella vinosa ***	**CBS 117.14**	**Scotland**	**LT963770**	**LT963509**
* Dendryphiella eucalyptorum *		CBS 137987 (T)	Spain	KJ869139	KJ869196
* Dendryphiella fasciculata *		MFLUCC 17-1074 (T)	Thailand	MF399213	MF399214
*** Dendryphiella paravinosa ***	*** Dendryphiella vinosa ***	**CBS 118716**	**New Zealand**	**LT963357**	**LT963359**
*** Dendryphiella paravinosa ***	*** Dendryphiella vinosa ***	**CBS 121797**	**Spain**	**LT963354**	**LT963355**
* Dendryphiella paravinosa *		CBS 141286 (T)	Italy	KX228257	KX228309
*** Dendryphiella variabilis ***	*** Dendryphiella vinosa ***	**CBS 584.96 (T)**	**Cuba**	**LT963453**	**LT963454**
* Dendryphiella vinosa *		NBRC 32669	Japan	DQ307316	03266901^3^
* Dendryphiella vinosa *		–	–	–	EU848590
* Dictyocheirospora bannica *		KH 332 (T)	Japan	LC014543	AB807513
* Dictyocheirospora pseudomusae *		KH 412	Japan	LC014549	AB807516
* Dictyocheirospora rotunda *		MFLUCC 14-0293b (T)	Thailand	KU179099	KU179100
* Dictyosporium bulbosum *		yone 221	Japan	LC014544	AB807511
* Dictyosporium elegans *		NBRC 32502 (T)	Japan	DQ018087	DQ018100
* Dictyosporium strelitziae *		CBS 123359 (T)	South Africa	FJ839618	FJ839653
* Digitodesmium bambusicola *		CBS 110279 (T)	Philippines	DQ018091	DQ018103
* Gregarithecium curvisporum *		KT 922 (T)	Japan	AB809644	AB807547
* Jalapriya inflata *		NTOU 3855	UK	JQ267362	JQ267363
* Jalapriya pulchra *		MFLUCC 15-0348 (T)	China	KU179108	KU179109
* Jalapriya toruloides *		CBS 209.65	–	DQ018093	DQ018104
*** Neodendryphiella mali ***	*** Diplococcium asperum ***	**CBS 139.95 (T)**	**Italy**	**LT906655**	**LT906657**
*** Neodendryphiella mali ***	***Dendryphiella* sp.**	**FMR 17003**	**Spain**	**LT993734**	**LT993735**
*** Neodendryphiella michoacanensis ***	***Dendryphiella* sp.**	**FMR 16098 (T)**	**Mexico**	**LT906660**	**LT906658**
*** Neodendryphiella tarraconensis ***	***Dendryphiella* sp.**	**FMR 16234 (T)**	**Spain**	**LT906659**	**LT906656**
* Paradendryphiella arenaria *		CBS 181.58 (T)	France	KF156010	KC793338
* Paradendryphiella salina *		CBS 142.60	United Kingdom	DQ411540	KC793339
* Pseudocoleophoma calamagrostidis *		KT 3284 (T)	Japan	LC014592	LC014609
* Pseudocoleophoma polygonicola *		KT 731 (T)	Japan	AB809634	AB807546
* Pseudodictyosporium elegans *		CBS 688.93 (T)	Taiwan	DQ018099	DQ018106
* Pseudodictyosporium wauense *		NBRC 30078	Japan	DQ018098	DQ018105
*** Torula herbarum ***	*** Dendryphiella infuscans ***	**CBS 381.81**	**Netherlands**	**LT963446**	**LT963455**

**^1^**CBS: Culture collection of the Westerdijk Fungal Biodiversity Institute, Utrecht, the Netherlands; FMR: Facultat de Medicina, Universitat Rovira i Virgili, Reus, Spain; HMAS: The Mycological Herbarium of the Chinese Academy of Science; KH: K. Hirayama; KT: K. Tanaka; MFLUCC: Mae Fah Luang University Culture Collection; NBRC: NITE Biological Resource Centre, Japan; NTOU: Institute of Marine Biology, National Taiwan Ocean University; RK: R. Kodsueb; yone: H. Yonezawa. (T): ex-type strain.

**^2^** Sequences newly generated in this study are indicated in bold.

**^3^** Number of sequence of the NBRC database.

### DNA extraction, sequencing and phylogenetic analysis

The isolates were cultured on PDA for 7 days at 25 °C in darkness. The DNA was extracted through the modified protocol of Werner et al. (1998). The primer pairs ITS5/ITS4 and NL1/NL4b were used to amplify ITS regions, including the 5.8S gene and the D1/D2 domain of the LSU of the nrDNA, respectively, following Cano et al. (2004). PCR products were purified and stored at -20 °C until sequencing. The same pairs of primers were used to obtain the sequences at Macrogen Europe (Macrogen Inc. Amsterdam, The Netherlands). Finally, the sequences were assembled and edited using SeqMan v. 7.0.0 (DNAStar Lasergene, Madison, WI, USA) to obtain the consensus sequences.

The sequences generated in the present study were compared with those of the National Center for Biotechnology Information (NCBI) using the Basic Local Alignment Search Tool (BLAST). Alignments for each locus were made with the MEGA (Molecular Evolutionary Genetics Analysis) software v. 6.0. (Tamura et al. 2013), using the ClustalW algorithm (Thompson et al. 1994) and refined with MUSCLE (Edgar 2004) or manually, if necessary, on the same platform. The alignment included our sequences complemented with available sequences of NCBI and NITE Biological Resource Center (NBRC) of species that conformed the different genera of the family Dictyosporiaceae (Table 1). This determined the phylogenetic position of the dendryphiella-like isolates in this group of fungi. Phylogenetic reconstructions with ITS and LSU sequences were made using Maximum Likelihood (ML) and Bayesian Inference (BI) approaches under the MEGA software v. 6.0. (Tamura et al. 2013) and MrBayes v. 3.2.6 (Ronquist et al. 2012), respectively.

For the ML phylogenetic analysis of the LSU region, the best nucleotide substitution model determined by the same programme was the Kimura 2-parameter with Gamma distribution and, for the ITS region, it was the General Time Reversible model with Gamma distribution. The combined analysis of these two phylogenetic markers was tested through Incongruence Length Difference (ILD) implemented in the Winclada programme (Farris et al. 1994). For the combined analysis of LSU and ITS sequences, the best nucleotide substitution model was the General Time Reversible with Gamma distribution and Invariant sites (G+I). ML bootstrap values (BML) ≥70% were considered significant.

For the BI phylogenetic analysis, the best nucleotide substitution model was determined using jModelTest (Posada 2008). For the LSU region, we used the Kimura 2-parameter with Gamma distribution (K80+G) and, for the ITS symmetrical model, we used Gamma distribution (SYM+G). The parameter settings used were two simultaneous runs of 5M generations, four Markov chains, sampled every 1000 generations. The 50% majority-rule consensus tree and posterior probability values (PP) were calculated after discarding the first 25% of the samples. A PP value of ≥0.95 was considered significant.

The DNA sequences and alignments generated in this study were deposited in GenBank (Table 1) and in TreeBASE (http://treebase.org), respectively.

### Phenotypic study

The microscopic characterisation of the fungi studied was carried out according to Marin-Felix et al. (2017), using autoclaved pine twig arranged on the surface of water agar (PNA) after 7 days at 25 °C in darkness. Measurements and descriptions of the structures were taken from the specimens mounted in Shear’s solution. Photomicrographs were obtained using a Zeiss Axio-Imager M1 light microscope (Zeiss, Oberkochen, Germany) with a DeltaPix Infinity × digital camera.

Macroscopic characterisation of the colonies was made on PDA, Oatmeal Agar (OA; Oatmeal 30 g, agar 13 g, distilled water 1 l), Potato Carrot Agar (PCA; potato 20 g, carrot 20 g, agar 13 g, distilled water 1 l), SNA (KH_2_PO_4_ 1 g, KNO 1 g, MgSO_4_×7H_2_O 0.5 g, KCl 0.5 g, Glucose 0.2 g, Sucrose 0.2 g, agar 14 g, distilled water 1 l) and Malt Extract Agar (MEA; Peptone 1 g, Glucose 20 g, Malt Extract 20 g, agar 15 g, distilled water 1 l) after 14 days at 25 °C in darkness. Colony colours in descriptions were matched with Kornerup and Wanscher (1978). Cardinal temperatures for growth were obtained on PDA after 14 days in darkness.

Nomenclatural novelties and descriptions were deposited in MycoBank (Crous et al. 2004). Ex-type cultures and holotypes, which consisted of dried cultures, were deposited at the CBS. Additionally, living cultures of the new species were also preserved in the Faculty of Medicine in Reus (FMR, Spain).

## Results

The BLAST query revealed that LSU sequences of our dendryphiella-like isolates (FMR 16098, FMR 16234 and FMR 17003) showed a high percentage of identity (99%) with that of the isolate CBS 139.95 of *Di.asperum* and all of them were related to the Dictyosporiaceae. However, they showed a sequence identity of between 96-97% with LSU sequences of *Dictyosporium* species and other members of this family, including several species of *Dendryphiella* deposited in the GenBank. The ITS sequences did not match significantly any of those deposited in the NCBI database.

**Figure 1.**
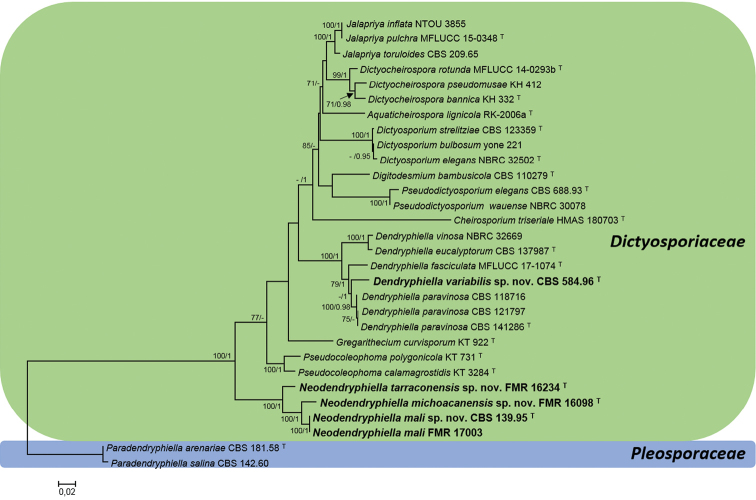
Maximum Likelihood (ML) tree constructed with the ITS and LSU sequences of 30 strains representatives of different taxa in the families Dictyosporiaceae and Pleosporaceae. The phylogenetic tree was rooted with *Paradendryphiellaarenaria* and *P.salina*. Bootstrap support values for ML greater than 70% and Bayesian posterior probabilities greater than 0.95 are given near nodes, respectively. Names of species newly described here are indicated in bold. Branch lengths are proportional to distance. ^T^ Ex-type strain.

We carried out individual and combined analyses with the LSU and ITS loci to assess relationships with members of the Dictyosporiaceae, including reference strains of *D.vinosa* and *D.infuscans* sequenced in the present study. Single phylogenies of LSU and ITS loci encompassed 31 and 30 sequences, respectively, representing 12 genera and including *Paradendryphiellaarenaria* and *P.salina* (Pleosporaceae) as outgroup (Figs S1 and S2 in the supplementary material). LSU analysis comprised 630 bp from which 111 bp were variable and 84 bp phylogenetically informative. The ITS comprised 496 bp, 266 bp being variable and 206 bp being phylogenetically informative. The topology of trees for single loci were very similar and the ILD test showed that the LSU and ITS datasets loci were congruent (P = 0.16) and could be combined. The final combined analysis encompassed 30 sequences and comprised 1126 bp (ITS 496 bp, LSU 630 bp). The ML tree showed that FMR 16098, FMR 16234, FMR 17003 and CBS 139.95 clustered together in a well-supported undescribed monophyletic lineage representing a new genus in the family (Fig. 1). The LSU and ITS sequence comparison of the four isolates revealed them as different taxa. The low identity values together with the morphological differences found amongst them allow us to propose three new species in this new genus, which are described below.

Regarding the five *Dendryphiella* strains included in this study, only three (CBS 118716, CBS 121797 and CBS 854.96) nested in the well-supported clade of *Dendryphiella* and none of them matched sequences representative of the type species of the genus *D.vinosa* (DQ 307316.1, EU848590.1 and NBRC-03266901) and used previously by other authors to establish the relationship of *D.vinosa* with the Dictyosporiaceae (Gareth Jones et al. 2008, Crous et al. 2014, 2016, Tanaka et al. 2015, Boonmee et al. 2016, Liu et al. 2017). The strains CBS 118716 and CBS 121797 matched the ex-type strain of *D.paravinosa* (CBS 141286); while CBS 584.96 nested in a terminal subclade with *D.fasciculata* and *D.paravinosa*, but it was placed in a single branch representative of a distinct taxa (Fig. 1). Its genetic difference and the production of conidia with up to 7 septa, a distinct morphological feature with respect to the accepted species of *Dendryphiella* (Liu et al. 2017, Hyde et al. 2018), justify the proposal of a new species in this genus. The other two isolates that had been received as *Dendryphiella* did not belong to this genus. The oldest reference strain of *D.vinosa* (CBS 117.14) corresponded to *Drechslerabiseptata* and the strain previously identified as *D.infuscans* (CBS 381.81) matched *Torulaherbarum*. The molecular identification of all the isolates included in this study is provided in Table 1.

## Taxonomy

### Neodendryphiella

Taxon classificationFungiPleosporalesDictyosporiaceae

Iturrieta-González, Dania García & Gené gen. nov.

824664

#### Etymology.

The name refers to the morphological similarity with *Dendryphiella*.

#### Type species.

*Neodendryphiellatarraconensis* Iturrieta-González, Gené & Dania García.

#### Description.

*Conidiophores* semi-macronematous to macronematous, mononematous, erect or slightly flexuous, unbranched or branched towards the apical region, septate, subhyaline to brown, smooth to verrucose, cylindrical, some slightly swollen in the conidiogenous loci. *Conidiogenous* integrated, terminal or intercalary, polytretic, cylindrical or clavate, forming conidia in acropetal branched chains. *Ramoconidia* aseptate or septate, pale brown, smooth to verruculose, mostly cylindrical or subcylindrical, rounded apex and truncate base, with several pores and conidial scars often thickened and darkened. *Conidia* blastocatenate, aseptate or septate, pale brown, verruculose to verrucose, ellipsoidal, doliiform, clavate or subcylindrical, with scars thickened and darkened. *Sexual morph* not observed.

#### Distribution.

Italy, Mexico and Spain.

### Neodendryphiella mali

Taxon classificationFungiPleosporalesDictyosporiaceae

Iturrieta-González, Gené & Dania García sp. nov.

824665

Fig. 2

#### Etymology.

Name refers to the substrate, *Malusdomestica*, where the type strain of the species was collected.

#### Type.

Italy, Dipt. Prot. Valor. Agroalimentare, from leaf of *Malusdomestica*, Feb. 1995, A. Cesari (holotype CBS H-23477, culture ex-type CBS 139.95).

#### Description.

*Mycelium* superficial and immersed, composed of septate, branched, smooth to verruculose, hyaline to pale brown hyphae of 1–3 μm wide. *Conidiophores* semi-macronematous to macronematous, mononematous, erect or slightly flexuous, branched or unbranched, up to 11-septate, cylindrical, up to 385 μm long, 3–4 μm wide, brown, usually darker toward the base, smooth to verrucose. *Conidiogenous* terminal and intercalary, mostly cylindrical, 8–38 × 3–4(–5) μm, with 1–4 pores. *Ramoconidia* 0–1-septate, with up to 3 terminal and lateral pores, pale brown, smooth to verruculose, mostly cylindrical, (11–)15–17(–21) × 3–4 μm. *Conidia* catenate, with up to 10 conidia in the terminal unbranched part, (0–)1-septate, usually not constricted at the septum, pale brown, verruculose to verrucose, ellipsoidal, doliiform or subcylindrical with more or less rounded ends, 4–15 × 3–5 μm.

**Figure 2.**
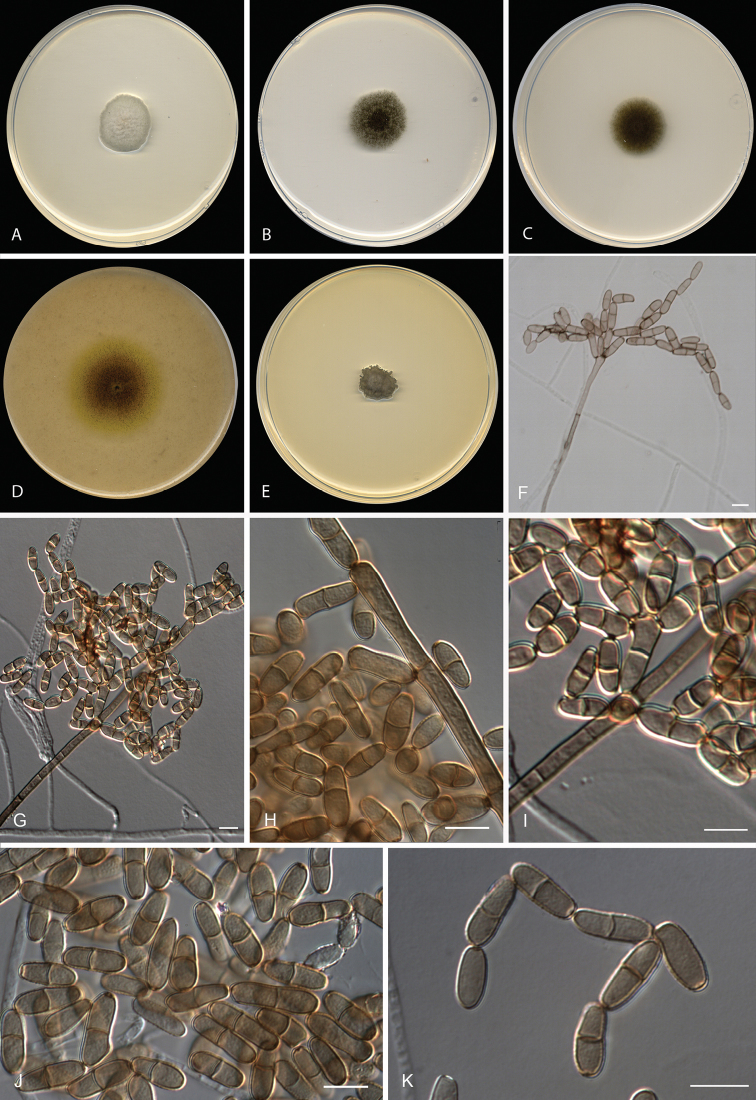
*Neodendryphiellamali* sp. nov. (ex-type CBS 139.95). **A–E** Colonies on **A**PDA**B** PCA **C** SNA **D** OA **E** MEA at 25 °C after 14 d **F–K** Conidiophores and conidia. Scale bars: 10 µm (**F–K**).

#### Culture characteristics

(14 d at 25 °C). Colonies on PDA reaching 22 mm diam., convex, slightly convoluted at the centre, pastel grey to white (1C1/1A1), aerial mycelium scarce, with slightly fimbriate margin; reverse olive brown to yellowish-brown (4D3/3A2). On PCA attaining 23 mm diam., flat, olive brown to greyish-beige (4F8/4C2), aerial mycelium scarce, slightly fimbriate margin; reverse greyish-beige to brownish-grey (4C2/4D2). On OA reaching 40 mm diam., flat, granular, yellowish-brown to reddish-yellow (5E8/4B7), aerial mycelium scarce, with a regular margin; reverse olive brown to yellowish-brown (4D8/4B7). On SNA attaining 24 mm diam., flat, slightly granular, olive brown to grey (4F8/4B1), aerial mycelium scarce, with fimbriate margin; reverse yellowish-brown (5F7/5E4). On MEA reaching 11–15 mm diam., umbonate, slightly cerebriform towards the periphery, velvety, olive grey (3E2), with irregular margin; reverse olive grey (3E2).

#### Cardinal temperature for growth.

Optimum 25 °C, maximum 30 °C, minimum 10 °C.

#### Distribution.

Italy and Spain.

#### Additional isolates examined.

Spain, Els Ports de Beseit Natural Park, Teruel, from herbivore dung, Oct. 2017, Dania García (FMR 17003)

#### Notes.

Although LSU sequences of *N.mali* (CBS 139.95 and FMR 17003) were very similar to those of *N.michoacanensis* (FMR 16098) and *N.tarraconensis* (FMR 16234), ITS regions showed a similarity of 96.2% (identities = 441/458, gaps 2/458 (0 %)) with respect to *N.michoacanensis* and of 92.3% (identities = 423/458, gaps 1/458 (0%)) with respect to *N.tarraconensis*. ITS sequences of the two latter species described below were 92.1% similar (identities = 422/458, gaps 0/458 (0%)).

*Neodendryphillamali* is morphologically very similar to *N.michoacanensis* since both have conidia and ramoconidia 0–1-septate; however, *N.michoacanensis* has shorter conidiophores (up to 280 μm long) and terminal conidial branches with fewer conidia (up to 4 per branch), which measure 5–16(–18) × 3–6 μm. In addition, 2-septate conidia can also be present in *N.michoacanensis* and this species tends to grow faster than *N.mali* on PDA (34 mm vs 22 mm diam. after 14 d, respectively) and PCA (42 mm vs 23 mm diam. after 14 d, respectively). *Neodendryphiellamali* also resembles *D.infuscans*, but the latter exhibits longer conidiophores, up to 500 μm and smooth to minutely verruculose conidia with up to 2 septa (Ellis 1971). However, the protologue of *D.infuscans* (as *Cladosporiuminfuscans*; Thümen 1879), which was based on a specimen collected in Aiken (USA), describes conidia 0–1-septate, smooth-walled and up to 10 μm long. No living culture of the type specimen was preserved for further comparison.

As mentioned before, the strain CBS 139.95 was identified as *Di.asperum* and found by other authors to be related with dictyosporium-like fungi (Shenoy et al. 2010, Tanaka et al. 2015). However, the protologue of *Di.asperum* was characterised by single or fasciculate conidiophores, which were up to 250 μm long, bearing terminal or subterminal, short and unbranched chains of conidia with only 1 septum (Pirozynski 1972), morphological features that do not fit with those observed in the above-mentioned strain. We therefore concluded that it was a misidentified strain and clearly represents a different species. At any rate, it is of note that the taxonomy of *Di.asperum* is controversial because of the different interpretation of the morphological features of Pirozynski’s specimen (DAOM 133941c isotype). Holubová-Jechová (1982) described conidiogenous cells showing inconspicuous denticles or conidiogenous scars instead of the typical pores in conidiogenous cells of *Diplococcium* and suggested excluding this species from the genus. On the other hand, Goh and Hyde (1998) re-examined the isotype of *Di.asperum* and observed the typical pores of tretic conidiogenesis, considering it an acceptable species for *Diplococcium*. However, since only herbarium material is preserved for comparison (Pirozynski 1972), its phylogeny remains uncertain.

### Neodendryphiella michoacanensis

Taxon classificationFungiPleosporalesDictyosporiaceae

Iturrieta-González, Dania García & Gené sp. nov.

824666

Fig. 3

#### Etymology.

Name refers to Michoacán, the geographical area where the fungus was collected.

#### Type.

Mexico, Michoacán, Villa Jiménez, from soil, Sept. 2016, E. Rodriguez-Andrade (holotype CBS H-23478; culture ex-type CBS 144323 = FMR 16098).

#### Description.

*Mycelium* superficial and immersed, composed of septate, branched, smooth to verruculose and hyaline to pale brown hyphae of 1–3 μm wide. *Conidiophores* semi-macronematous to macronematous, mononematous, erect or slightly flexuous, slightly branched, 1–13 septate, cylindrical or slightly swollen in the conidiogenous loci, 44–280 × 2–4 μm, brown, usually darker toward the base, smooth or verruculose, verrucose at the base. *Conidiogenous* terminal and intercalary, cylindrical or clavate, 11–62 × 3–5 μm, with up to 3 pores. *Ramoconidia* (0–)1-septate, with up to 4 terminal or subterminal pores, pale brown, smooth to verruculose, cylindrical, subcylindrical, to slightly clavate, with more or less rounded apex and truncate base, 12–23 × 3–4(–5) μm. *Conidia* catenate, with up to 4 conidia in the terminal unbranched part, (0–)1(–2)-septate, some slightly constricted at the septum, pale brown, verruculose to verrucose, ellipsoidal or subcylindrical, 5–16(–18) × 3–6 μm.

**Figure 3.**
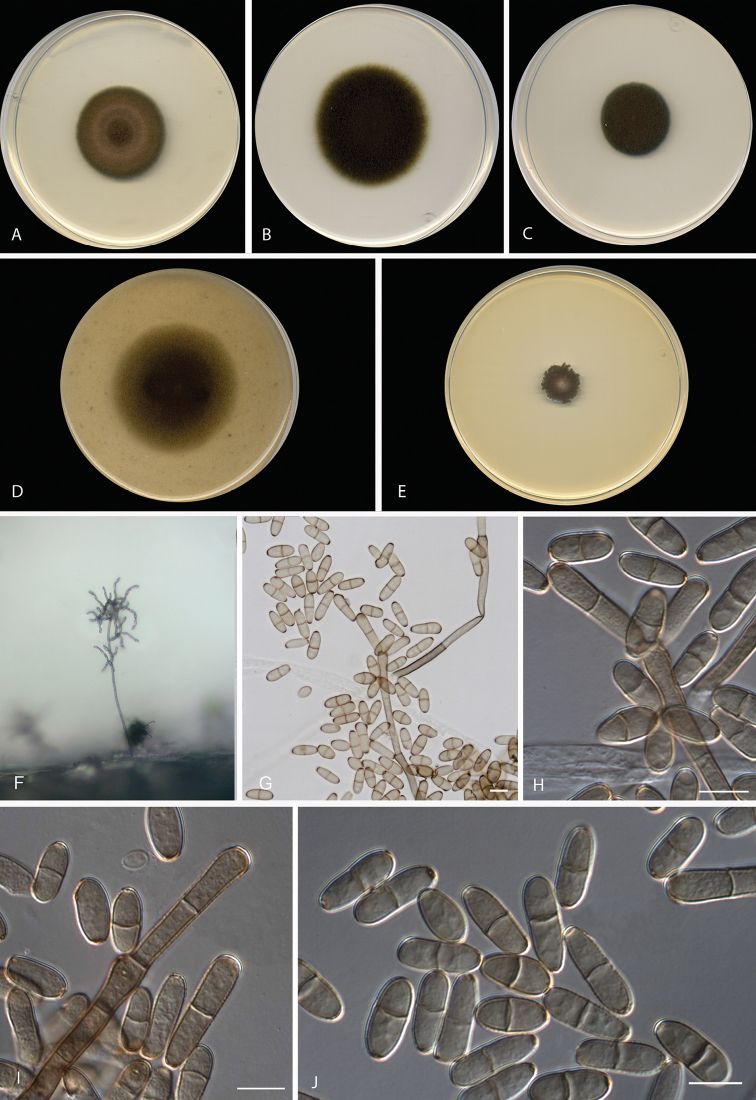
*Neodendryphiellamichoacanensis* sp. nov. (ex-type FMR 16098). **A–E** Colonies on **A**PDA**B** PCA **C** SNA **D** OA **E** MEA at 25 °C after 14 d **F–J** Conidiophores and conidia. Scale bars: 10 µm (**G–J**).

#### Culture characteristics

(14 d at 25 °C). Colonies on PDA reaching 34 mm diam., slightly umbonate, velvety, olive brown (4F6/4E8), with slightly fimbriate margin; reverse dark green (30F8) to black. On PCA attaining 42 mm diam., flat, granular, olive brown (4F8), aerial mycelium scarce, fimbriate margin; reverse dark green to olive brown (30F8/4F8). On OA reaching 48 mm diam., flat, granular, yellowish-brown to olive (5F4/3D4), aerial mycelium scarce, with a regular margin; reverse brownish-grey to greyish-yellow (4D2/3B6). On SNA attaining 22 mm diam., flat, slightly granular, olive brown (4F8), aerial mycelium scarce, with slightly fimbriate margin; reverse dark green (30F8) to black. On MEA reaching 13–15 mm diam., slightly umbonate, flat towards the periphery, velvety, yellowish-grey to olive (3C2/3F8), with white irregular margin; reverse olive grey to dark green (3E2/30F8).

#### Cardinal temperature for growth.

Optimum 25 °C, maximum 30 °C, minimum 10 °C.

#### Distribution.

México.

#### Notes.

*Neodendryphiellamichoacanensis* morphologically resembles *N.mali*, in its conidiogenous apparatus with 0–1-septate ramoconidia, but the latter differs by having longer conidiophores (up to 385 μm), terminal conidial chains with up to 10 conidia and its conidia are 0–1-septate and smaller (4–15 × 3–5 μm). *Neodendryphiellamichoacanensis* also resembles *D.uniseptata* in their conidial morphology, but ramoconidia of the latter species are often aseptate and can be up to 30 μm long (Matsushima 1971). *Dendryphiellauniseptata* is only known from the type material, which was collected in Honiara (Japan) and no ex-type culture was preserved. This species was considered a synonym of *D.infuscans* by Matsushima (1975) but not accepted by Liu et al. (2017).

### Neodendryphiella tarraconensis

Taxon classificationFungiPleosporalesDictyosporiaceae

Iturrieta-González, Gené & Dania García sp. nov.

824667

Fig. 4

#### Etymology.

Name refers to Tarragona, the geographical area where the fungus was collected.

#### Type.

Spain, Tarragona, from garden soil, Feb. 2017, I. Iturrieta-González (holotype CBS H-23479, culture ex-type CBS 144324 = FMR 16234).

#### Description.

*Mycelium* superficial and immersed abundant, composed of septate, branched, smooth to verruculose, hyaline to pale brown hyphae, 1–2 μm wide. *Conidiophores* macronematous, mononematous, erect or slightly flexuous, branched or unbranched, up to 6-septate, cylindrical, 19–185 × 2–5 μm, brown, smooth, darker and finely verrucolose towards the base. *Conidiogenous* terminal and intercalary, subcylindrical to clavate, 9–35 × (2–)3–4(–5) μm, with up to 2 pores. *Ramoconidia* (0–)1–2(–3)-septate, usually slightly constricted at the septa, with up to 3 terminal and subterminal pores, pale brown, smooth to verruculose, mostly cylindrical, with rounded apex and truncate base, 12–21(–23) × 4–5 μm. *Conidia* catenate, with up to 7 conidia in the terminal unbranched part, (0–)1–2-septate, pale brown, verruculose, ellipsoidal or subcylindrical with more or less rounded ends, 6–21 × 3–6(–7) μm; when 1-septate, the septum is often submedial and slightly constricted, when 2-septate, usually constricted at only one septum.

**Figure 4.**
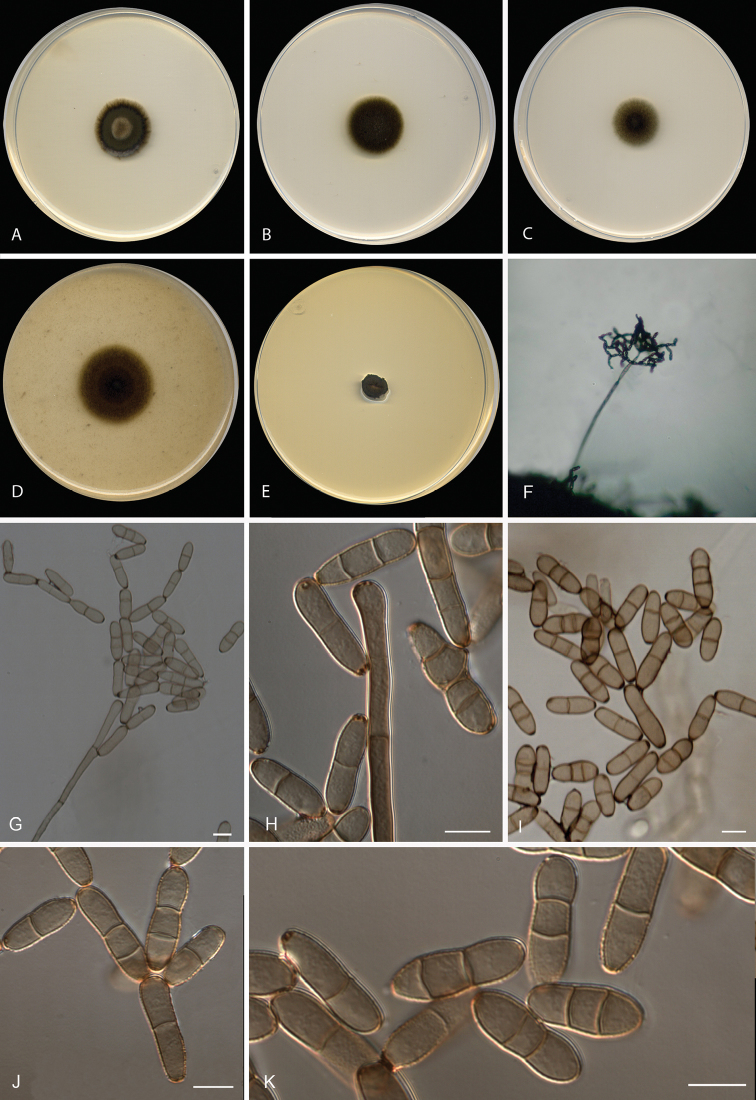
*Neodendryphiellatarraconensis* sp. nov. (ex-type FMR 16234). **A–E** Colonies on **A**PDA**B** PCA **C** SNA **D** OA **E** MEA at 25 °C after 14 d **F–K** Conidiophores and conidia. Scale bars:10 µm (**G–K**).

#### Culture characteristics

(14 d at 25 °C). Colonies on PDA reaching 23 mm diam., umbonate, velvety, greyish-brown to olive brown (5E3/4F8), with slightly fimbriate margin; reverse dark green (30F8) to black. On PCA attaining 24 mm diam., flat, velvety, olive brown (4F8), slightly fimbriate margin, reverse dark green to olive brown (28F5/3B2) with a pale yellow (4A3) diffusible pigment. On OA reaching 30 mm diam., flat, slightly granular, yellowish-brown to olive brown (5F8/4F4), aerial mycelium scarce, with regular margin; reverse yellowish-brown to olive brown (5F8/4F4). On SNA attaining 21 mm diam., flat, slightly granular, yellowish-brown to olive (5F4/3F5), aerial mycelium scarce, with fimbriate margin; reverse yellowish-brown to olive (5F4/3F5). On MEA reaching 8-10 mm diam., slightly elevated but depressed at the centre, radially folded, velvety, olive (2F8), with irregular margin; reverse olive (2F4).

#### Cardinal temperature for growth.

Optimum 25 °C, maximum 30 °C, minimum 10 °C.

#### Distribution.

Spain.

#### Notes.

In addition to the genetic differences mentioned above, *N.tarraconensis* differs from the other two species in the genus by the presence of ramoconidia with up to 3 septa and conidia from terminal branches with mostly 1–2-septate. It is noteworthy that 1-septate conidia usually show a slightly longer basal cell since the septum is submedial and, when 2-septate, often only one of the septa is constricted, features not described in any species of *Dendryphiella* and *Neodendryphiella*.

### Dendryphiella variabilis

Taxon classificationFungiPleosporalesDictyosporiaceae

Iturrieta-González, Dania García & Gené sp. nov.

824668

Fig. 5

#### Etymology.

Name refers to the variable number of septa in the conidia.

#### Type.

Cuba, from a dead leaf of a Lauraceous tree, 1996, R.F. Castañeda (holotype CBS H-23476; ex-type cultures CBS 584.96 = INIFAT C95/105-4 = MUCL 39840 = FMR 16563).

#### Description.

*Mycelium* superficial and immersed, composed of septate, branched, smooth to verruculose hyaline to pale brown hyphae, 1–3 μm wide. *Conidiophores* macronematous, mononematous, often arranged in loose fascicules, erect or slightly flexuous, branched, 1–8-septate, nodulose toward the apex, up to 143 μm long, 2–6 μm wide, brown, smooth to verruculose. *Conidiogenous* terminal and intercalary, sympodially extended towards the apex, with 1–5 pores surrounded by a thickened and darkened wall, clavate, 7–37 × 3–6(–7) μm. *Ramoconidia* (0–)2–3-septate, cylindrical to subcylindrical, with rounded ends, 16–27 × 5–6 μm, usually with 2 apical pores, conidial scars thickened and darkened. *Conidia* in short branched chains, with up to 5 conidia in the terminal unbranched part, (0–)3(–7)-septate, some constricted at the medial septum, pale brown, verruculose to verrucose, cylindrical or subcylindrical, with rounded ends, 6–44 × 4–6 μm, conidial scars often thickened and darkened. *Sexual morph* not observed.

**Figure 5.**
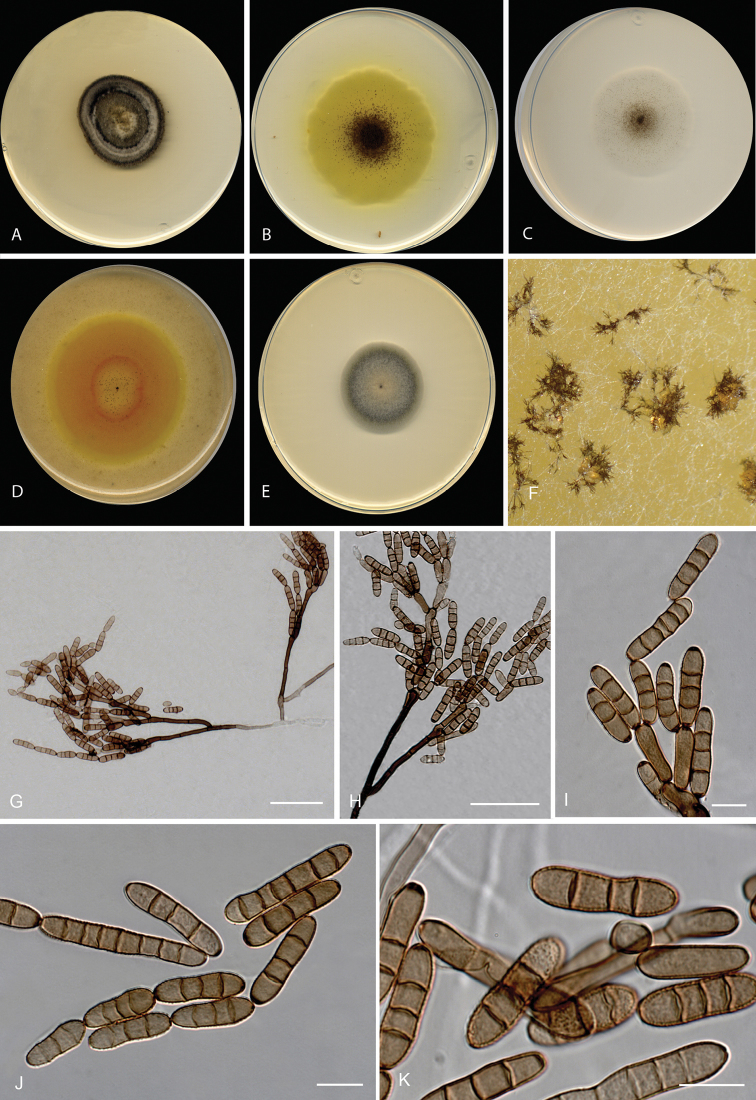
*Dendryphiellavariabilis* sp. nov. (ex-type CBS 584.96). **A–E** Colonies on **A**PDA**B** PCA **C** SNA **D** OA **E** MEA at 25 °C after 14 d **F** Exudates and conidiophores produced on OA **G–K** Conidiophores and conidia. Scale bars: 50 µm (**G–H**), 10 µm (**I–K**).

#### Culture characteristics

(14 d at 25 °C). Colonies on PDA reaching 30–33 mm diam., slightly umbonate, flat towards the periphery, velvety, irregularly coloured yellowish-grey to olive brown (4B2/4D3) and brownish-grey to yellowish-brown (5F2/5F4), with irregular margin; reverse yellowish-brown (5F8) to black. On PCA attaining 48 mm diam., flat, granular to velvety, yellowish-brown (5F8), aerial mycelium scarce, undulate margin; reverse olive to greyish-yellow (3F4/3B4), with a pale yellow diffusible pigment. On OA reaching 58 mm diam., flat, slightly granular, blond to reddish-yellow (5C4/4A7), light yellow (4A4) at the periphery, aerial mycelium scarce, with a regular margin, with scarce pale brown exudate; reverse same colouration with the colony surface. On SNA attaining 40 mm diam., flat, slightly granular to velvety, yellowish-brown to grey (5F7/4B1), with fimbriate margin; reverse brownish-grey to white (5D2/1A1). On MEA reaching 32 mm diam., flat, cottony, yellowish-grey to olive (4B2/3F4), yellowish-grey (3B2) at the periphery, with regular margin; reverse dark green to white (30F8/1A1).

#### Cardinal temperature for growth.

Optimum 25 °C, maximum 30 °C, minimum 15 °C.

#### Distribution.

Cuba.

#### Notes.

*Dendryphiellavariabilis* differs from *D.paravinosa* mainly by having longer conidia (up to 44 μm), which can have up to 7 septa. The conidia of *D.paravinosa* are up to 3-septate and measure (10−)24−27(−33) × (6−)7(−7.5) μm (Crous et al. 2016). The only species of the genus reported with conidia up to 5-septate are *D.eucalyptorum* and *D.vinosa*, but they are smaller, measuring (19−)20−23(−25) × 5(−7) μm in the former (Crous et al. 2014) and 13−39 × 4−8 μm in the latter (Ellis 1971). The other closely related species to *D.variabilis* is *D.fasciculata* (Fig. 1), but it mainly differs by the presence of fasciculate conidiophores and 3-septate conidia (Liu et al. 2017).

## Discussion

The present study proposes the genus *Neodendryphiella* based on the analysis of the ITS and LSU sequences, which represented an undescribed monophyletic lineage related but phylogenetically distant from the morphologically similar genus *Dendryphiella*. Both genera belong to the Dictyosporiaceae (Dothideomycetes) and share similar conidiophore morphology with polytretic conidiogenous cells forming usually septate conidia arranged in acropetal branched chains. *Dendryphiella* can be differentiated by the presence of nodulose conidiophores and conidiogeneous cells with pores surrounded by a thickened and darkened wall, which are absent in *Neodrendryphiella*. Other genera of the Dothideomycetes, although accommodated in different orders or families with a similar conidiogenous apparatus are *Dendryphion* (Toluraceae, Pleosporales) (Crous et al. 2014, Crous et al. 2015), *Dendryphiopsis* (Kirschsteiniotheliaceae, Kirschsteiniotheliales) (Su et al. 2016, Hernández-Restrepo et al. 2017) and *Paradendryphiella* (Pleosporaceae, Pleosporales) (Woudenberg et al. 2013). However, the genus *Diplococcium* in Leotiomycetes also shows similar asexual propagules (Shenoy et al. 2010, Hernández-Restrepo et al. 2017), which complicates the classification of these fungi based exclusively on morphological features.

Our phylogenetic study not only allowed us to distinguish very similar isolates in three distinct species, *N.mali, N.michoacanensis* and *N.tarraconensis*, but also helped us to correctly identify some strains that had previously been attributed to *Dendryphiella* (Table 1). In addition, it is of note that, considering the species accepted in *Dendryphiella* (Liu et al. 2017, Hyde et al. 2018), this genus seems to be morphologically heterogeneous and probably polyphyletic. It includes species with apparently polyblastic denticulate conidiogenous cells, such as *D.eucalypti* (Matsushima, 1983) or *D.uniseptata* (Matsushima, 1971), rather than polytretic conidiogenous cells typical of *Dendryphiella* (Rao and Narania 1974, Crous et al. 2014, 2016) or species that produce solitary conidia, such as *D.cruzalmensis* (Batista, 1946) or *D.lycopersicifolia* (Batista & Peres, 1961). In this scenario, therefore, *Dendryphiella* requires a further taxonomic re-evaluation. However, taking into account that only herbarium material is available for the type *D.vinosa* (preserved in the Kew herbarium, as *Helminthosporiumvinosum*) there is a need to re-collect this species from the type locality (Cuba) for epitypification and giving nomenclature stability to the genus.

## Supplementary Material

XML Treatment for Neodendryphiella

XML Treatment for Neodendryphiella mali

XML Treatment for Neodendryphiella michoacanensis

XML Treatment for Neodendryphiella tarraconensis

XML Treatment for Dendryphiella variabilis

## Supplementary materials

Supplementary material 1 *Neodendryphiella* gen. nov. Tree LSU This is an open access article distributed under the terms of the Creative
Commons Attribution License (CC BY 4.0), which permits unrestricted use, distribution,
and reproduction in any medium, provided the original author and source are
credited.Isabel Iturrieta-González, Josepa Gené, Josep Guarro, Rafael F. Castañeda-Ruiz, Dania García

Supplementary material 2 *Neodendryphyella* gen. nov. Tree ITS This is an open access article distributed under the terms of the Creative
Commons Attribution License (CC BY 4.0), which permits unrestricted use, distribution,
and reproduction in any medium, provided the original author and source are
credited.Isabel Iturrieta-González, Josepa Gené, Josep Guarro, Rafael F. Castañeda-Ruiz, Dania García
