# Supplementary figures and images for: Neodendryphiella, a novel genus of the Dictyosporiaceae (Pleosporales)
Source: MycoKeys. 2018 Jul 26;(37):19–38. doi: 10.3897/mycokeys.37.27275 (PMC6108305; doi:10.3897/mycokeys.37.27275)

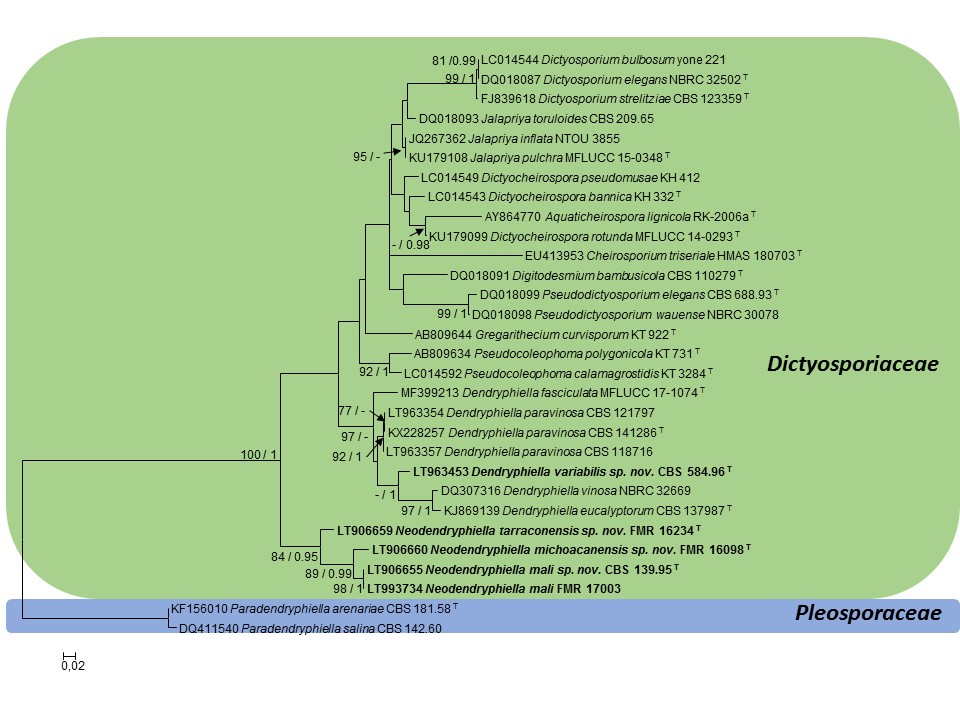

Supplement: Supplementary material 1 — Neodendryphiella gen. nov. Tree LSU [file mycokeys-37-019-s002.jpg]
